# Using a Double-Coil TMS Protocol to Assess Preparatory Inhibition Bilaterally

**DOI:** 10.3389/fnins.2018.00139

**Published:** 2018-03-08

**Authors:** Pierre Vassiliadis, Julien Grandjean, Gerard Derosiere, Ysaline de Wilde, Louise Quemener, Julie Duque

**Affiliations:** Institute of Neuroscience, Université Catholique de Louvain, Brussels, Belgium

**Keywords:** transcranial magnetic stimulation, motor-evoked potentials, primary motor cortex, corticospinal excitability, coefficient of variation, action preparation, inhibition

## Abstract

Transcranial magnetic stimulation (TMS) applied over the primary motor cortex (M1), elicits motor-evoked potentials (MEPs) in contralateral limb muscles which are valuable indicators of corticospinal excitability (CSE) at the time of stimulation. So far, most studies have used single-coil TMS over one M1, yielding MEPs in muscles of a single limb—usually the hand. However, tracking CSE in the two hands simultaneously would be useful in many contexts. We recently showed that, in the resting state, double-coil stimulation of the two M1 with a 1 ms inter-pulse interval (double-coil_1 ms_ TMS) elicits MEPs in both hands that are comparable to MEPs obtained using single-coil TMS. To further evaluate this new technique, we considered the MEPs elicited by double-coil_1 ms_ TMS in an instructed-delay choice reaction time task where a prepared response has to be withheld until an imperative signal is displayed. Single-coil TMS studies have repetitively shown that in this type of task, the motor system is transiently inhibited during the delay period, as evident from the broad suppression of MEP amplitudes. Here, we aimed at investigating whether a comparable inhibitory effect can be observed with MEPs elicited using double-coil_1 ms_ TMS. To do so, we compared the amplitude as well as the coefficient of variation (CV) of MEPs produced by double-coil_1 ms_ or single-coil TMS during action preparation. We observed that MEPs were suppressed (smaller amplitude) and often less variable (smaller CV) during the delay period compared to baseline. Importantly, these effects were equivalent whether single-coil or double-coil_1 ms_ TMS was used. This suggests that double-coil_1 ms_ TMS is a reliable tool to assess CSE, not only when subjects are at rest, but also when they are involved in a task, opening new research horizons for scientists interested in the corticospinal correlates of human behavior.

## Introduction

Transcranial magnetic stimulation (TMS), a technique used to assess corticospinal excitability (CSE), has gained substantial attention since it was first described about 30 years ago (Ziemann, 2017). The amplitude of motor-evoked potentials (MEPs) elicited in muscles of the contralateral limb (often the hand) by TMS over the primary motor cortex (M1) is a precious indicator of CSE at the time of stimulation (Bestmann and Krakauer, 2015; Bestmann and Duque, 2016; Duque et al., 2017). Comparing MEP amplitudes in different conditions have helped to characterize the corticospinal correlates of various neural processes including those underlying action preparation and stopping (Duque et al., 2010, 2012, 2013; van den Wildenberg et al., 2010; Greenhouse et al., 2012; Majid et al., 2012; Quoilin and Derosiere, 2015), decision making and reward processing (Klein et al., 2012; Klein-Flügge and Bestmann, 2012; Cos et al., 2014; Zénon et al., 2015; Derosiere et al., 2017a,b), sustained attention (Derosière et al., 2015), speech (Labruna et al., 2011b; Neef et al., 2015), and motor imagery (Ruffino et al., 2017). TMS has also proved useful in characterizing the corticospinal correlates of behavioral deficits in several neurologic disorders (Badawy et al., 2012) including stroke (Auriat et al., 2015; Stinear et al., 2015; Smith and Stinear, 2016; Boddington and Reynolds, 2017), Parkinson's disease (Valls-Solé et al., 1994; Lefaucheur, 2005; Soysal et al., 2008; Benninger and Hallett, 2015), or Alzheimer's disease (Guerra et al., 2011).

To date, almost all TMS-based CSE studies have recorded MEPs from muscles of a single hand following the application of TMS over one M1 only. Hence, in most experiments, the MEP data have only provided researchers with half of the story, increasing the probability of seeing data being misinterpreted. This occurs because applying TMS over both M1 in separate blocks doubles the duration of the experiment, making it impossible to fit all the conditions in a single session. For example, studies investigating inhibitory processes during action preparation have typically recorded MEPs from left hand muscles (following right M1 TMS) in instructed-delay choice RT tasks where subjects have to withhold cued left or right hand responses (e.g., left or right index finger key-presses) until an imperative signal is displayed (Duque and Ivry, 2009; Duque et al., 2010; Greenhouse et al., 2015b; Lebon et al., 2016; Quoilin et al., 2016): left MEPs are deeply suppressed in this context (compared to a baseline), a phenomenon often referred to as preparatory inhibition (Duque et al., 2017). Critically, many studies have reported a stronger left MEP suppression in conditions where the target muscle is selected for the forthcoming movement (i.e., left response) compared to when it is non-selected (i.e., right response) and it has been commonly accepted that this difference results from the distinct function (selected vs. non-selected) of the left hand muscle in these two situations (Duque et al., 2010, 2014; Labruna et al., 2014). That is, preparatory inhibition is thought to be more prominent for selected than non-selected effector representations. Yet, there is a substantial confound here because besides the function (selected vs. non-selected), conditions also differ in regard to the hand being cued for the response (left vs. right). Hence, the stronger left MEP suppression with left than right hand responses may be due to the use of the non-dominant vs. dominant hand rather than to the distinct function of the targeted muscle in these trials.

Recently, we have proposed the use of double-coil TMS over both M1, to obtain MEPs from bilateral muscles at once (Wilhelm et al., 2016; Grandjean et al., 2018). In these previous studies, we tested a double-coil TMS method where the two M1 are stimulated with a 1 ms inter-pulse interval (double-coil_1 ms_ TMS). An interval between the two TMS pulses is necessary to avoid direct electromagnetic interference between the two stimulating coils. Yet, the latter must be kept short enough to avoid cortical interactions through the corpus callosum occurring with delays as small as 4 ms (Ferbert et al., 1992; Hanajima et al., 2001; reviewed in Reis et al., 2008). In Grandjean et al. (2018), MEPs elicited using this new double-coil_1 ms_ approach (MEP_double_) were recorded for five different intensities of stimulation while participants were completely relaxed, at rest, and were compared to those elicited in the same conditions using single-coil TMS (MEP_single_) applied successively over the two M1. Note that given the 1 ms inter-pulse interval in double-coil_1 ms_ trials, MEP_double_ were either evoked by a 1st (MEP_double−1_) or a 2nd (MEP_double−2_) TMS pulse. Importantly, the study revealed that MEP_double−1_ and MEP_double−2_ are comparable to MEP_single_ when elicited at rest, regardless of the TMS intensity, suggesting that this method may be used to assess CSE bilaterally. However, it still remains to be determined whether double-coil_1 ms_ TMS produces comparable MEPs as single-coil TMS in the context of a task.

In the present study, we compared MEP_double−1&2_ and MEP_single_ during action preparation, applying double-coil_1 ms_ or single-coil TMS in an instructed-delay choice RT task where subjects have to withhold a cued response until an imperative signal is displayed (Bestmann and Duque, 2016; Quoilin et al., 2016; Duque et al., 2017). We compared the strength of preparatory inhibition when probed using double-coil_1 ms_ or single-coil TMS. Some of these results have already been reported in abstract form (Grandjean et al., 2017a,b).

## Materials and methods

### Participants

A total of 15 right-handed healthy subjects participated in the present study (*n* = 15; 10 women; 22.4 ± 1.63 years old). Handedness was determined via a shortened version of the Edinburgh Handedness inventory Oldfield (1971) and all subjects filled out a TMS safety questionnaire. None of the participants suffered from any neurological disorder or had a history of psychiatric illness, drug or alcohol abuse; neither was anybody undergoing a drug treatment that could influence their performance or their neural activity. All subjects were financially compensated for their participation and provided written informed consent. The protocol was approved by the Ethics Committee of the Université Catholique de Louvain.

### The “rolling ball” task

Participants sat in front of a 21-inch monitor screen positioned about 60 cm in front of them with their arms semi-flexed and both hands resting palm-down on a response device developed in our laboratory (Quoilin et al., 2016). They performed an instructed-delay choice reaction time (RT) task, which required them to choose between abduction movements of the left or right index finger. The task was implemented with Matlab 7.5 (the Mathworks, Natick, Massachusetts, USAS) using the Psychophysics Toolbox extensions (Brainard, 1997; Pelli, 1997). The refresh rate of the monitor was set at 100 Hz.

**Figure 1:**
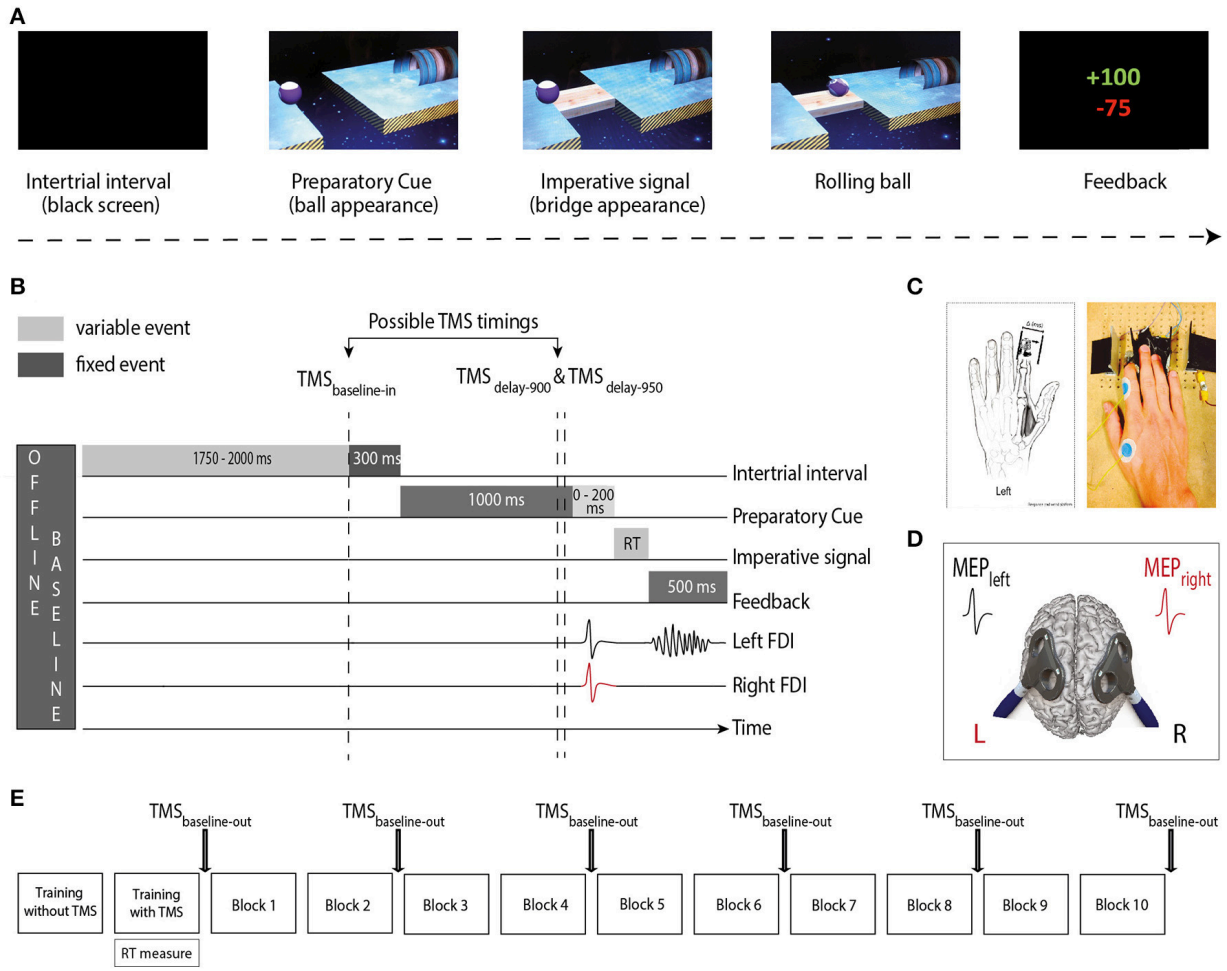
**(A)** “Rolling Ball” task. Subjects were asked to choose between responding with the left or right index finger according to the position of a ball (Preparatory cue) appearing on the left or right part of the screen (left in the current example). They had to wait until the onset of a bridge (Imperative signal) to release their response. The ball then rolled on the bridge (when the subjects answered correctly) to reach a goal located on the other side of the gap. A feedback reflecting how fast and accurate the subjects had been concluded each trial. **(B)** Time course of a trial. Each trial started with a blank screen (intertrial interval; 2,050–2,300 ms). Then, the preparatory cue appeared for a variable delay period (1,000–1,200 ms), followed by the imperative signal until the reaction time (RT). The feedback was presented at the end of each trial for 500 ms. TMS pulses occurred either during the intertrial interval (1,750–2,000 ms after the blank screen onset; TMS_baseline−in_), or during the delay period (900 or 950 ms after the preparatory cue onset; TMS_delay−900_ and TMS_delay−950_). In Double-coil_1 ms_ trials, motor-evoked potentials (MEPs) were elicited in the first dorsal interosseous (FDI) of both hands at a near simultaneous time (1 ms delay); in single-coil trials, MEPs were elicited in the left or right hand. The figure displays a left hand trial with double-coil_1 ms_ at TMS_delay−950_. **(C)** The response device. Index finger responses were recorded using a home-made device positioned under the left (graphic representation) and right (photographic representation) hands **(D)** TMS protocol. Two figure-eight-shaped coils were placed over the subject's primary motor cortex (M1), eliciting MEPs in the left and/or right FDI. **(E)** Time-course of the experiment. After two training blocks (see section Materials and Methods), subjects executed 10 blocks of 40 trials during which MEPs were elicited at TMS_baseline−in_ or TMS _delay_; MEPs were also elicited outside the blocks (TMS_baseline−out_), before block 1 and after blocks 2, 4, 6, 8, and 10.

The task consisted in a virtual “Rolling Ball” game previously used in another study (Quoilin et al., 2016; Figure 1A). In this game, participants were informed that the position of a preparatory cue (i.e., a ball separated from a goal by a gap) indicated the movement side for the forthcoming response: if the ball was on the left side of the screen, subjects had to prepare a left index finger response (to get ready to “shoot the ball into the goal”) and if the ball was on the right side, subjects had to prepare a right index finger response. Subjects were explicitly told to withhold their response until the onset of an imperative signal (i.e., a bridge). The latter appeared 1,000–1,200 ms after the ball and remained on the screen until a finger movement was detected or for a maximum duration of 500 ms. When the bridge was on the screen, subjects had to respond as fast as possible to allow the ball to roll on it and to quickly reach the goal. Subjects knew that they would get a score after each trial reflecting how fast and accurate they had been on the previous trial. Note that in each block, some catch trials (trials in which the bridge did not appear; 5% of all trials) were included. Subjects were required not to respond on these trials and were penalized if they did so. Hence, they had to avoid initiating their response prematurely, before the bridge onset. Trials were separated by the presentation of a blank screen lasting for a duration that varied between 2,050 and 2,300 ms (Figure 1B).

The home-made response device (Figure 1C) was composed of two pairs of metal edges fixed on a wooden platform (one for each hand) and each trial of the Rolling Ball game required participants to move one index finger from the outer to the inner metal edge (i.e., abduction of the index finger). The contact between the finger and the metal parts of the device was continuously monitored using a Makey Makey printed circuit board with an ATMega32u4 microcontroller running the Arduino Leonardo firmware, based on the principle of high resistance switching between two electrical contacts. This device provided us with a very precise measure of the RTs (precision = 1 ms) and allowed us to control for any anticipated movement. That is, the device permanently checked the initial position of each index finger (which had to be in contact with the outer metal edge) and any contact release before the onset of the imperative signal led to the cancellation of the trial and to a penalty.

Subjects received a feedback of their performance at the end of each trial. On correct trials, the feedback score (displayed in green) was inversely proportional to the reaction time (RT): the faster the subjects, the higher the score. The RT was defined as the time interval between the onset of the bridge and the time when the index finger left the outer metal edge of the response device. The score was determined based on the following equation, with ∝ = 0, 8 *median RT* measured at the end of the training session just before the main experiment:

$$x=\frac{\left(100.\left(\propto \right)\right)}{\left(\propto +{\left(\frac{\text{RT}-\propto }{10}\right)}^{2,4}\right)}$$

Using this equation, scores on correct trials ranged from 1 to 100. Incorrect responses were penalized with negative scores displayed in red. They involved responses occurring too early, referred to as “anticipation errors” (penalized by 75 points), responses occurring too late, referred to as “time-out errors” (penalized by 50 points), responses provided with the incorrect hand (penalized by 20 points), referred to as “choice errors” and responses provided on catch trials (penalized by 12 points), referred as “catch errors.” Anticipation errors consisted in responses provided either before the bridge onset or after its onset but with a RT smaller than 100 ms. Time-out errors consisted in responses provided in more than 500 ms (after the bridge offset). Note that when subjects succeeded not to respond on a catch trial, they were rewarded by +12 points. The total score was always displayed at the end of each block.

### TMS protocol

TMS was delivered through one or two small figure-of-eight shaped coils (wing internal diameter 35 mm), each connected either to a Magstim 200^2^ magnetic stimulator (Magstim, Whitland, Dyfed, UK) or a Magstim Bistim^2^ magnetic stimulator. Both stimulators delivered monophasic pulses and their relationship to a specific hemisphere was counterbalanced between subjects. Each coil was placed tangentially over one primary motor cortex (M1) with the handle pointing backward and laterally at a 45° angle away from the midline, approximately perpendicular to the central sulcus (Figure 1D). Small coils were chosen because in most subjects, it is not possible to place two large coils over the two M1s at the same time. For each M1, the optimal scalp position to elicit a contralateral MEP in the first dorsal interosseous muscle (FDI) was identified and marked on a head cap placed on the subject's scalp to provide a reference mark throughout the experiment (Duque et al., 2014, 2016; Klein et al., 2014). Importantly, this was done by always checking for the fact that the two coils could be positioned simultaneously on the head without touching each other; to reduce electromagnetic interference it was sometimes necessary to adjust the orientation of the coils a little but these adaptations remained marginal and did not preclude us from obtaining the best MEP amplitudes.

The resting Motor Threshold (rMT) was determined at the hotspot for each M1 as the minimal TMS intensity required to evoke MEPs of about 50 μV peak-to-peak in the relaxed FDI muscle in at least 5 out of 10 consecutive trials. Across participants, the rMTs corresponded to 41.7 ± 5.05 and 40.8 ± 6.39% of the maximum stimulator output for the left and the right FDI, respectively. The intensity of TMS used throughout the experiment was always set at 115% of the individual rMT for each hemisphere.

### Experimental procedure

The experiment started with two training blocks. The first one (20 trials) was conducted without TMS whereas the second one (40 trials) involved TMS, exactly as in the main experiment. Thereby, the subjects could first practice the task without being disturbed by the TMS pulse and then get used to the stimulations while performing the task in the second training block. The latter block also served to obtain the median RTs, used to individualize the scores on correct trials (see below). Then, during the main phase of the experiment, subjects performed 10 blocks of 40 trials (Figure 1E). Using these numbers, we obtained 20 MEPs in each condition.

The goal of the present experiment was to compare the amplitude of MEPs elicited during motor preparation using either single-coil or double-coil_1 ms_ TMS. In half of the trials, single-coil TMS was used, eliciting MEPs in a single hand (MEP_single_), either in the left or the right FDI in a balanced proportion. In the other half, MEPs were elicited in both hands at once (MEP_double_) using a double-coil_1 ms_ method where the two M1 are stimulated with a 1 ms inter-pulse interval (double-coil_1 ms_; Grandjean et al., 2018). In all subjects, half of the double-coil_1 ms_ trials involved a pulse over left M1 first whereas the other half of the trials involved a pulse over the right M1 first. Therefore, for each hand, MEPs_double_ could either result from a first (MEP_double−1_) or a second pulse (MEP_double−2_). Importantly, the single- and double-coil_1 ms_ trials were always randomized within a block so that the subject could not anticipate the type of pulse (single or double) they would have next, an aspect that could bias MEPs, as suggested in a previous study (Wilhelm et al., 2016).

Single- and double-coil_1 ms_ TMS pulses were applied at three different timings during the Rolling Ball task (only one pulse per trial; Figure 1B). First, some TMS pulses occurred during the intertrial interval, at a random time falling 1,750–2,000 ms after the blank screen onset; these trials were used to compare MEP_single_ and MEP_double_ at baseline (rest) within the blocks (TMS_baseline−in_, 20% of all trials). In the remaining trials, the TMS was delivered during the delay period either 900 ms (TMS_delay−900_, 40% of all trials) or 950 ms (TMS_delay−950_, 40% of all trials) after the occurrence of the preparatory cue. Based on previous studies (reviewed in Duque et al., 2017), we assumed that at these TMS_delay_ timings, inhibitory changes would be substantial whether MEPs are elicited in a selected condition (e.g., left MEPs elicited in a left hand trial) or a non-selected condition (e.g., left MEPs elicited in a right hand trial). Finally, we also recorded baseline MEPs outside the blocks (TMS_baseline−out_), at six different times (before block 1 and after blocks 2, 4, 6, 8, and 10; 20 MEPs each). These MEPs provided us with a measure of CSE outside the context of the task, at complete rest. Moreover, the comparison of MEP_single_ and MEP_double_ at TMS_baseline−out_ allowed us to check whether we could replicate our previous observations (Grandjean et al., 2018).

### Electromyography (EMG) recording

EMG activity was recorded from surface electrodes (Neuroline, Medicotest, Oelstykke, Denmark) placed over the left and right FDI. MEPs recorded from these homonymous muscles offered a measure of CSE changes occurring in muscles that are involved in the task (whether selected or non-selected). Note that for all participants, stimulating the hotspot for the FDI also elicited reliable MEPs in the abductor digiti minimi (ADM), a pinkie abductor muscle which is irrelevant for the task. These MEPs were also considered in the present study. EMG data were collected for 1,000 ms on each trial, starting 300 ms before the TMS pulse. The EMG signals were amplified (x1,000), bandpass filtered online (10–500 Hz; NeuroloLog; Digitimer), and digitalized at 2,000 Hz for offline analysis.

Trials with background EMG activity (root mean square computed from −250 to −50 ms before the TMS pulse) exceeding 3 standard deviations (*SD*) around the mean were discarded for the following analyses. This was done to prevent contamination of the MEP measurements by significant fluctuations in background EMG (Duque et al., 2014, 2016; Klein et al., 2014). The remaining MEPs were classified according to the experimental condition within which they had been elicited. Trials in which subjects made an error were also removed from the data set; the task was easy so these trials remained rare and errors were not analyzed.

For each condition, we excluded trials with a peak-to peak MEP amplitude exceeding 3 *SD* around the mean. After screening the data for errors, background EMG activity and outliers, a total of 15.9 ± 2.7 trials per condition were left to evaluate CSE changes during action preparation. One subject had to be taken off the MEP analyses because we encountered a technical problem during the experiment (remaining *n* = 14 subjects).

### Statistical analyses

Analyses were carried out with the RStudio software (version 1.0.153., RStudio, Inc., Boston, MA). The assumptions of normality and homogeneity of variance were tested before analyses. All data were systematically tested for the sphericity assumption using Maunchley's tests. The Greenhouse–Geisser correction was used for sphericity when necessary.

#### Reaction time

The RT data were classified according to whether subjects performed a movement with the left or right index finger (Mvt_SIDE_: Mvt_left_ or Mvt_right_). In addition, trials were divided depending on the time of the TMS pulse (TMS_TIMING_: TMS_baseline−in_ or TMS_delay_; trials with TMS_delay−900_ and TMS_delay−950_ pooled together for the RT analysis). Finally, RTs were considered separately for trials in which double-coil_1 ms_ or single-coil_1 ms_ TMS was used and for the latter condition we also distinguished trials according to whether the responding hand corresponded to the one in which the MEP was elicited or not (MEP_CONDITION_: MEP_double_, MEP_single−Resp_, MEP_single−NonResp_). These data were analyzed using a two-way analysis of variance for repeated measures (ANOVA_RM_) with the factors Mvt_SIDE_, TMS_TIMING_, and MEP_CONDITION_.

#### MEP amplitude

Analyses considered three main types of MEPs (MEP_TYPE_ = MEP_single_, MEP_double−1_, and MEP_double−2_) elicited in the left or right hand (MEP_SIDE_ = MEP_left_, MEP_right_), at one of four different timings (TMS_TIMING_ = TMS_baseline−out_, TMS_baseline−in_, TMS_delay−900_, and TMS_delay−950_), during preparation of a left or right side movement (Mvt_SIDE_ = Mvt_left_ or Mvt_right_).

In a first analysis, we focused on MEPs elicited at rest, when subjects were not preparing a response, considering both MEPs obtained outside the blocks (TMS_baseline−out_) and those acquired within the blocks (TMS_baseline−in_). These MEPs were log-transformed in order to normalize the data distribution. A three-way ANOVA_RM_ was then conducted on the normalized MEP data, with TMS_TIMING_ (TMS_baseline−out_ or TMS_baseline−in_), MEP_TYPE_ (MEP_single_, MEP_double−1_, or MEP_double−2_), and MEP_SIDE_ (MEP_left_ or MEP_right_) as within-subject factors.

Second, we aimed at comparing the strength of MEP suppression during the delay period according to whether a single- or double-coil_1 ms_ procedure was used. To do so, MEPs elicited at TMS_delay−900_ and TMS_delay−950_ were expressed in percentage of MEPs acquired at TMS_baseline−in_ for each condition. These data were log-transformed and multiple one-sided *t*-tests were performed to compare the MEPs elicited at TMS_delay−900_ and TMS_delay−950_ to a constant value of 2 [standing for the TMS_baseline−in_ MEPs because log(100) = 2]. In a second step, we analyzed these data using a four-way ANOVA_RM_ with TMS_TIMING_ (TMS_delay−900_ or TMS_delay−950_), MEP_TYPE_ (MEP_single_, MEP_double−1_, or MEP_double−2_), MEP_SIDE_ (MEP_left_ or MEP_right_), and Mvt_SIDE_ (Mvt_left_ or Mvt_right_) as within-subject factors.

In a further analysis, we assessed the statistical equivalence of MEP amplitudes elicited using a single-coil or double-coil_1 ms_ procedure. We did so by testing “average bioequivalence hypotheses” [Schuirmann, 1987; U.S. Food and Drug Administration, 2001; Luzar-Stiffler and Stiffler, 2002]; a procedure detailed in our previous study (Grandjean et al., 2018). Briefly, MEP_double−1_ and MEP_double−2_ elicited at TMS_baseline_ (TMS_baseline−in_ and TMS_baseline−out_) and TMS_delay_ (TMS_delay−900_ and TMS_delay−950_) were expressed as a percentage of MEP_single_ elicited at the same TMS_TIMING_. We then computed the log of the percentage obtained to further normalize the distribution of the data in each experimental condition. To be considered as equivalent to MEP_single_, the normalized data needed to be significantly different from the boundaries of a ±0.4 window centered around 2 (corresponding to a MEP_double_ data fitting within a ±20% window centered on 100% of MEP_single_ in log) [U.S. Food and Drug Administration, 2001; Luzar-Stiffler and Stiffler, 2002; Grandjean et al., 2018]. This was tested for each experimental condition, using two one-sided *t*-tests (one for each boundary) given our a priori hypotheses (Grandjean et al., 2018). In a second step, we also determined the smallest significant boundary for each condition. To do so, one-sided *t*-tests starting at ±0.4 around 2 (i.e., ±20% around 100% in log) and decreasing by ±0.02 (i.e., 1% of 2) were performed until we found the narrowest windows between which MEP_double−1_ and MEP_double−2_ significantly fitted (*p* < 0.05).

#### MEP coefficient of variation (CV)

The variability of MEP amplitudes was assessed by computing a coefficient of variation (CV = [*SD*/mean MEP amplitude] × 100) in each experimental condition (Klein-Flügge et al., 2013). Similar to the procedure followed for the analysis of MEP amplitudes, we first focused on CVs at rest (at TMS_baseline−out_ and TMS_baseline−in_; three-way ANOVA_RM_, same factors as for MEP amplitudes). Then, after having expressed the CVs at TMS_delay−900_ and TMS_delay950_ as a percentage of CVs at TMS_baseline−in_, we considered changes during the delay period (four-way ANOVA_RM_, same factors as for MEP amplitudes). The CVs were also log-transformed for these analyses as the data were not normally distributed. Finally, bioequivalence of CVs obtained in the context of double-coil_1 ms_ and single-coil TMS was also estimated for the TMS_baseline_ and TMS_delay_ timings, using the exact same procedure as for the MEP amplitudes.

*Post-hocs* comparisons were always conducted using the Fisher's Least Significant Difference (LSD) procedure. All of the data are expressed as mean ± SE and the significance level was set at *p* ≤ 0.05.

## Results

### Reaction time (RT)

**Figure 2:**
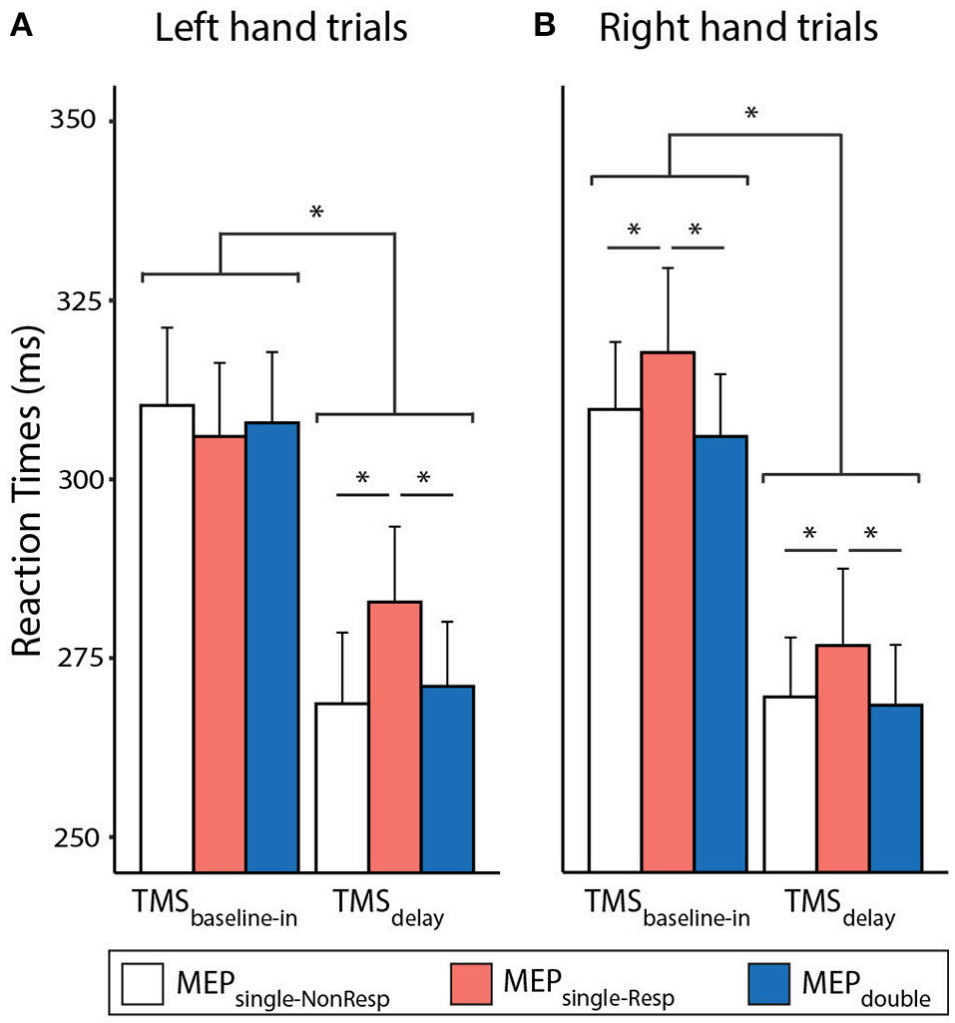
Left **(A)** and Right **(B)** hand reaction times (RTs, in ms) recorded in trials with TMS_baseline−in_ or TMS_delay_ (TMS_delay−900_ and TMS_delay−950_ pooled together), eliciting a MEP_single_ in the responding or non-responding hand (MEP_single−Resp_ or MEP_single−NonResp_, respectively) or MEP_double_ in both hands. ^*^Significantly different (*p* ≤ 0.05).

The RTs are shown on Figure 2 separately for the left and right hand trials. The ANOVA_RM_ revealed a significant influence of TMS_TIMING_ [*F*_(1,14)_ = 124.015 and *p* ≤ 0.001]: RTs were generally faster with TMS_delay_ (272.6 ± 36.4 ms) than with TMS_baseline−in_ (309.4 ± 38.8 ms), consistent with many previous reports showing that a TMS pulse applied close to the imperative signal can speed up the release of a motor response (Duque et al., 2012; Labruna et al., 2014; Greenhouse et al., 2015b). Furthermore, the MEP_CONDITION_ also influenced the RTs [*F*_(2, 28)_ = 6.007, *p* = 0.007]: Fisher LSD *post-hoc* tests revealed that RTs were significantly longer in the MEP_single−Resp_ condition than in the MEP_single−NonResp_ and MEP_double_ conditions (both *p* ≤ 0.004); the two latter were not different (*p* = 0.597). These results indicate that the RTs were slower in the presence of a single pulse eliciting a MEP in the responding hand compared to when the MEP was elicited in the non-responding hand or in both hands at once. Finally, the Mvt_SIDE_ × TMS_TIMING_ × MEP_CONDITION_ interaction was significant [*F*_(2, 28)_ = 5.125, *p* = 0.013]. As such, the slowing effect of MEP_single−Resp_ reported above was systematically observed with TMS_delay_ in both hands (all *p* ≤ 0.038). Yet, in trials with TMS_baseline−in_, it was only present for right hand (both *p* ≤ 0.023) but not for left hand trials (both *p* ≥ 0.198).

### MEP amplitude

#### FDI MEPs recorded at TMS_baseline_

**Figure 3:**
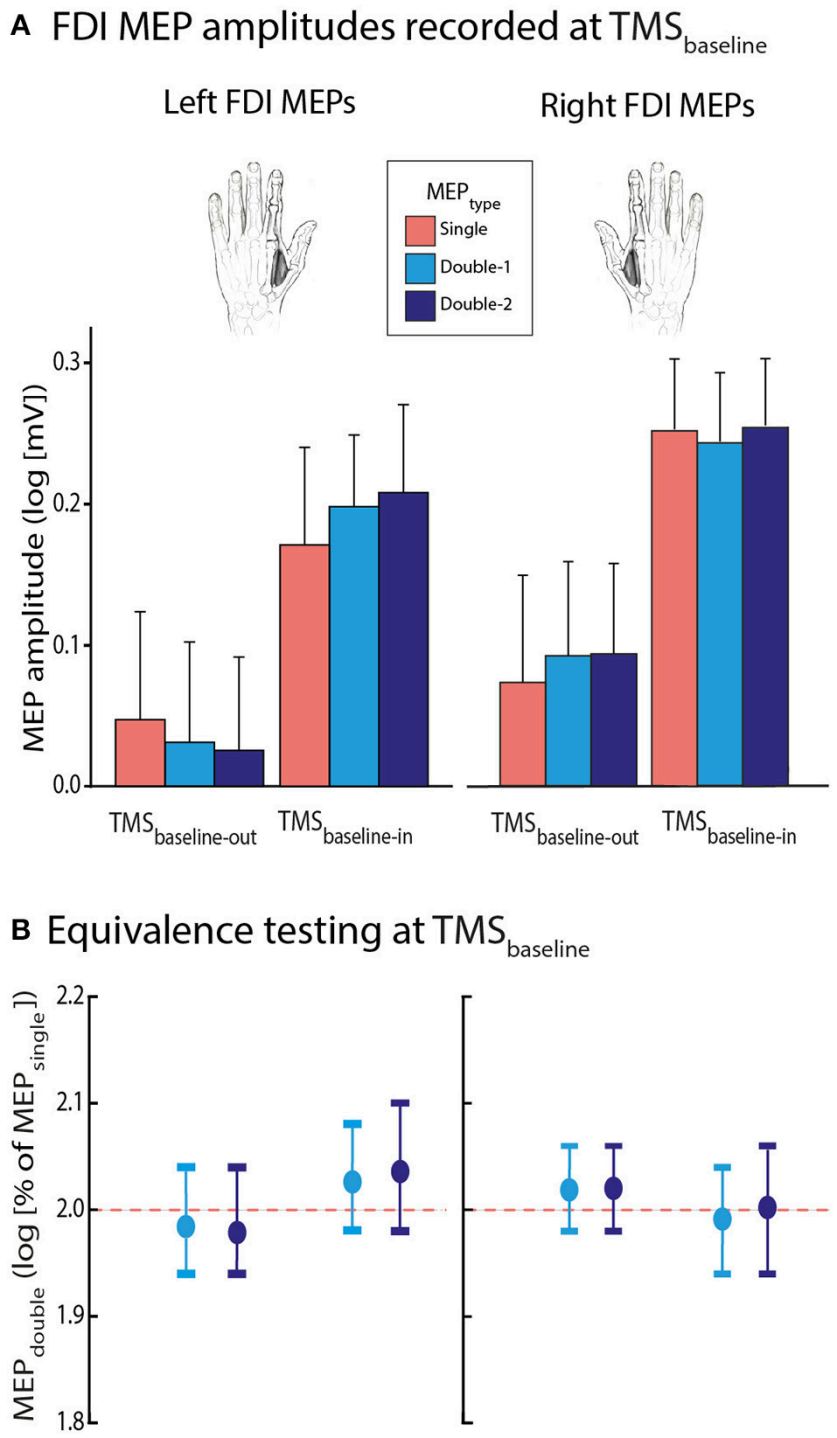
**(A)** Log-transformed MEP_single_ (red bars), MEP_double−1_ (light blue bars), and MEP_double−2_ (navy blue bars) at TMS_baseline−out_ and TMS_baseline−in_, elicited in the left or right first dorsal interosseous (FDI) muscle. Note that MEP amplitudes at TMS_baseline−in_ were significantly larger than at TMS_baseline−out_; *p* ≤ 0.001. **(B)** MEP_double−1_ and MEP_double−2_ amplitudes (expressed as log-transformed percentages of MEP_single_) significantly fitted in windows ranging between 1.94 and 2.10 l.u. [log(100) = 2], indicating comparable amplitudes for all MEP_TYPE_. The vertical bars represent the smallest significant boundaries around the mean for each condition. Each plot refers to the above color-coded condition on the x-axis.

First, we considered FDI MEPs acquired at rest, either during the blocks (TMS_baseline−in_) or outside them (TMS_baseline−out_). As evident on Figure 3A, MEPs were generally larger at TMS_baseline−in_ (1.8 ± 0.79 mV) than at TMS_baseline−out_ (1.3 ± 0.70 mV; *p* ≤ 0.001). Hence, MEP amplitudes were increased when elicited in the context of the task, as shown in previous reports (Labruna et al., 2011a; Klein et al., 2014; Duque et al., 2016). Importantly this increase was equivalent in all conditions and occurred in the same proportion whether MEPs were elicited using single-coil (MEP_single_) or double-coil_1 ms_ TMS (MEP_double−1_ and MEP_double−2_); the different MEP_TYPE_ never differed from one another, whether elicited at TMS_baseline−out_ or TMS_baseline−in_ [*F*_(2, 26)_ = 0.405, *p* = 0.671].

Second, we aimed to further assess the bioequivalence of the FDI MEP_TYPE_ at TMS_baseline_. To do so, similar to the procedure used in a previous study (Grandjean et al., 2018), we expressed the MEP_double−1_ and MEP_double−2_ data as a percentage of MEP_single_. We compared these percentages with boundaries set at ±20% around 100% (corresponding to MEP_single_), through multiple one-sided *t*-tests (Luzar-Stiffler and Stiffler, 2002). Notably, because the percentages were log-transformed for the analyses, this involved comparing them with boundaries set at ±0.4 around 2 log units (l.u) [because log(100) = 2]. At TMS_baseline−out_ as well as at TMS_baseline−in_, the log-transformed normalized MEP_double−1_ and MEP_double−2_ amplitudes significantly fitted into the ±0.4 window. As we can see on Figure 3B, the MEP_double−1_ and MEP_double−2_ even fitted in smaller windows (all MEP_double−1_ between 1.94 and 2.08 l.u.; i.e., between 97 and 104% of MEP_single_ and all MEP_double−2_ between 1.94 and 2.10 l.u. [97–105%], all *p* ≤ 0.05).

#### FDI MEPs recorded at TMS_delay_

**Figure 4:**
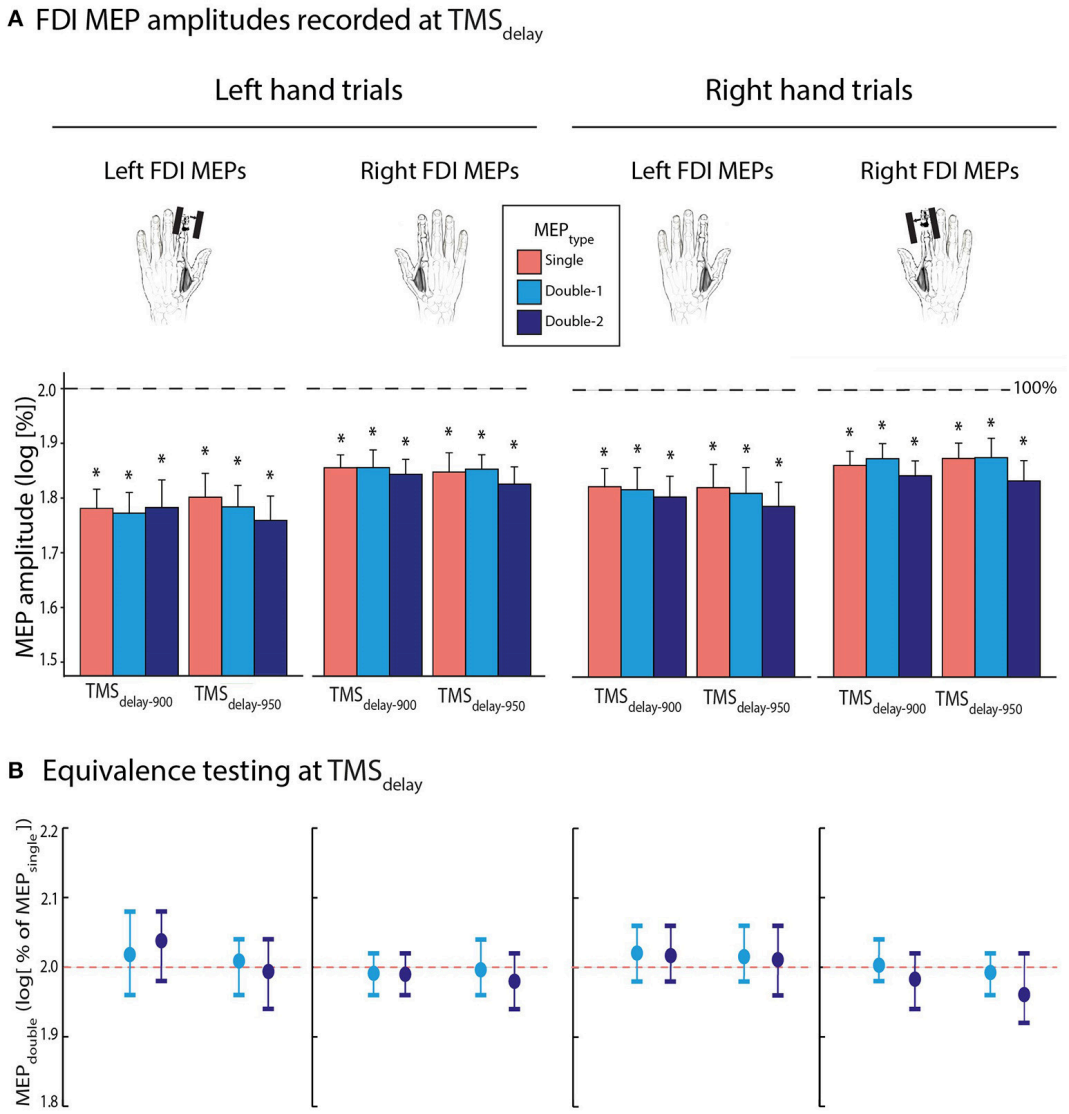
**(A)** Log-transformed MEP_single_ (red bars), MEP_double−1_ (light blue bars), and MEP_double−2_ (navy blue bars) at TMS_delay−900_ and TMS_delay−950_ (initially expressed as a percentage of TMS_baseline−in_), elicited in the left or right first dorsal interosseous (FDI). Data are shown separately for left (left panel) and right hand (right panel) trials. **(B)** Log-transformed MEP_double−1_ and MEP_double−2_ amplitudes at TMS_delay_ (initially expressed in percentage of MEP_single_). These data significantly fitted in windows ranging from 1.92 to 2.08 l.u. [i.e., between 96 and 104% of MEP_single_ in log], indicating comparable amplitudes for MEP_double_ and MEP_single_ during action preparation. The vertical bars represent the smallest significant boundaries around the mean for each condition. Each plot refers to the above color-coded condition on the x-axis. ^*^p ≤ 0.005.

Then, we evaluated FDI MEP amplitudes during action preparation. To do so, MEPs elicited at TMS_delay−900_ and TMS_delay−950_ were expressed as a percentage of MEPs elicited at TMS_baseline−in_. On average, MEPs equalled 69.7 ± 18.85 and 70.0 ± 21.13% of baseline when elicited at TMS_delay−900_ and TMS_delay−950_, respectively. These data were log-transformed for the analyses (Figure 4A); all normalized MEPs were smaller than 2 [i.e., log(100); all *t* ≤ −3.442, *p* ≤ 0.003], reflecting a consistent suppression of MEPs during the delay period, both at TMS_delay−900_ and TMS_delay−950_. Importantly, the ANOVA_RM_ did not reveal any significant effect of the factor MEP_TYPE_ [*F*_(2, 26)_ = 0.513, *p* = 0.685]: the MEPs acquired with double-coil_1 ms_ TMS, either by a first (MEP_double−1_) or second pulse (MEP_double−2_), were comparable to MEP_single_. Besides, MEP amplitudes were the same at both TMS_TIMING_ [*F*_(1, 13)_ = 0.115 and *p* ≥ 0.45] and did not depend on whether they were elicited in the left or right FDI [MEP_SIDE_: *F*_(1, 13)_ = 3.241, *p* = 0.095], or on whether they occurred during a left or right hand trial [Mvt_SIDE_: *F*_(1, 13)_ = 4.182, *p* = 0.062], although there was a small non-significant trend for the MEP suppression to be more pronounced preceding left hand trials, especially when probed in the left hand. None of the interactions were significant (all *F* ≤ 1.159, all *p* ≥ 0.330).

Concerning the bioequivalence testing at TMS_delay−900_ and TMS_delay−950_, the log-transformed MEP_double−1_ and MEP_double−2_ data (initially expressed in percentage of MEP_single_) significantly fitted into the ±0.4 window around 2. These data even fitted in smaller windows as shown on Figure 4B (all MEP_double−1_ between 1.96 and 2.08 l.u. [i.e., between 98 and 104% of MEP_single_] and all MEP_double−2_ between 1.92 and 2.08 l.u. [96–104%]; all *p* ≤ 0.05).

#### Additional analyses on FDI MEP amplitudes

We performed a three-way ANOVA_RM_ focusing on the normalized MEP_single_ data, with TMS_TIMING_ (TMS_delay−900_, TMS_delay−950_), MEP_SIDE_ (MEP_left_ or MEP_right_), and Mvt_SIDE_ (Mvt_left_ or Mvt_right_) as within-subject factors to ensure that the absence of effect between conditions in which the muscle was selected or not selected for the forthcoming response was not related to the inclusion of additional MEP_TYPES_ (MEP_double−1_ and MEP_double−2_). This ANOVA_RM_ did not reveal any significant MEP_SIDE_ x Mvt_SIDE_ interaction [*F*_(1,13)_ = 0.457, *p* = 0.511], neither did this interaction interact with the factor TMS_TIMING_ [TMS_TIMING_ x MEP_SIDE_ x Mvt_SIDE_: *F*_(1,13)_ = 1.99, *p* = 0.182]. Hence, the level of inhibition was comparable in selected and non-selected conditions in the present study, regardless of whether a single- or double-coil procedure was used.

#### Additional analyses on ADM MEP amplitudes

As mentioned above, stimulation of the hotspot for the FDI, also elicited MEPs in the ADM, a pinkie abductor. Although this muscle is irrelevant in the “Rolling Ball” game, its MEPs basically showed the same changes as those observed in the FDI, although in an attenuated manner. At rest, ADM MEPs were globally larger at TMS_baseline−in_ than TMS_baseline−out_ [*F*_(1, 13)_ = 24.791, *p* ≤ 0.001]. Most importantly, ANOVA_RM_ revealed that single-coil and double-coil_1 ms_ TMS elicited comparable ADM MEPs at rest [MEP_TYPE_ *F*_(2, 26)_ = 0.148, *p* = 0.863]. Consistently, the bioequivalence tests showed that all log-transformed MEP_double−1_ and MEP_double−2_ amplitudes (initially expressed in percentage of MEP_single_) significantly fitted into smaller windows than ±0.4 around 2: all MEP_double−1_ and MEP_double−2_ amplitudes fitted in a 1.92–2.08 window, i.e., 96–104%, all *p* ≤ 0.05).

In addition, ADM MEPs were also suppressed during the delay period (all *t* ≤ −2.042, all *p* ≤ 0.031), regardless of the TMS_TIMING_ [*F*_(1, 13)_ = 0.036, *p* = 0.853] or the MEP_SIDE_ [*F*_(1, 13)_ = 0.149, *p* = 0.705]. Note that the MEP suppression was significantly less pronounced preceding right than left hand movements [*F*_(1, 13)_ = 5.165, *p* = 0.041]. Importantly, the factor MEP_TYPE_ was non-significant [*F*_(2, 26)_ = 0.157, *p* = 0.855]. At both delay timings, all MEP_double−1_ and MEP_double−2_ amplitudes fitted in 1.94–2.08 [97–104%] and 1.92–2.06 [96–103%] windows, respectively. Thus the double-coil_1 ms_ protocol seemed to induce comparable MEPs as single-coil TMS in an irrelevant muscle as well.

### Coefficient of variation (CV) of MEPs

#### CV of FDI MEPs recorded at TMS_baseline_

**Figure 5:**
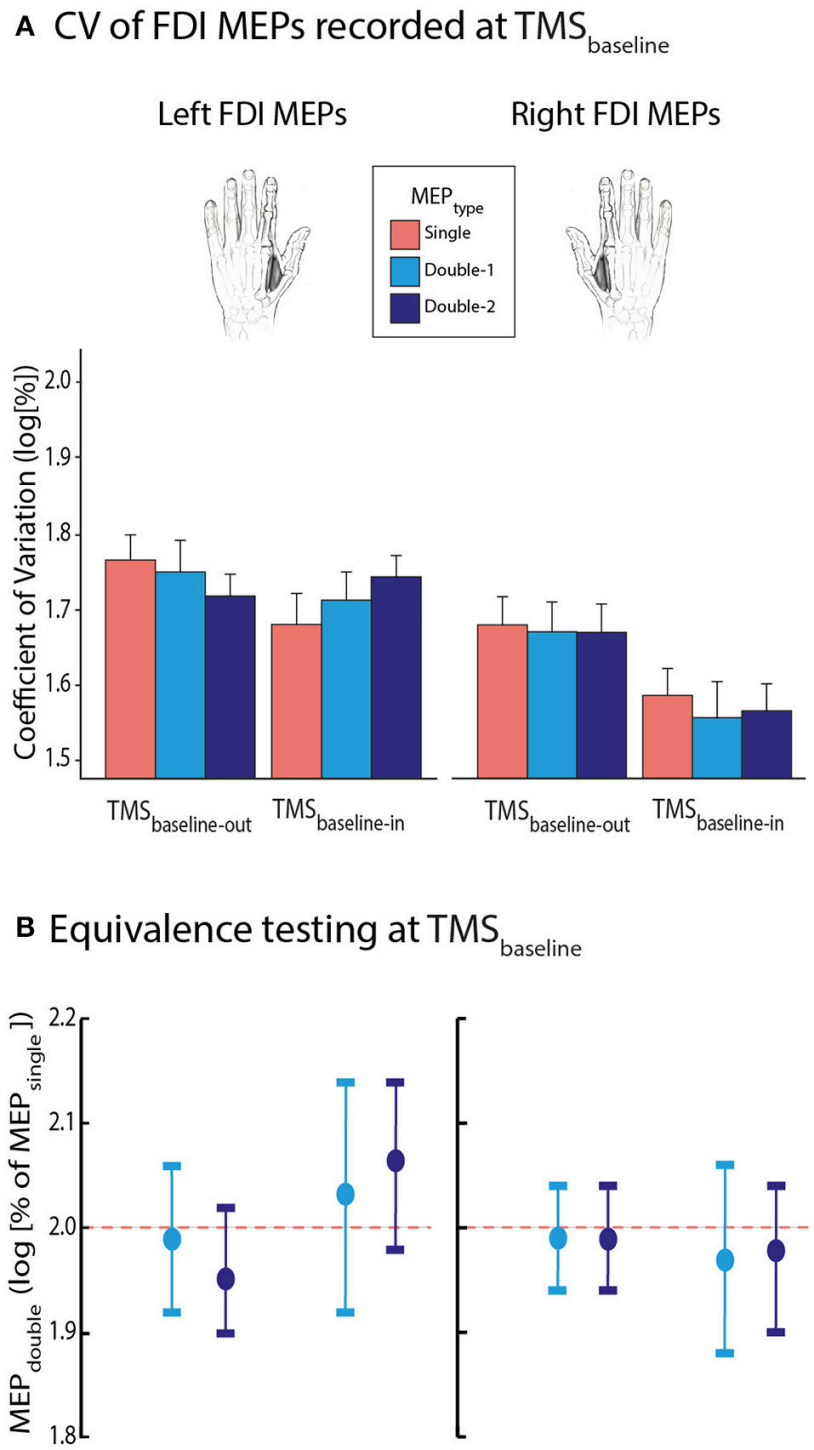
**(A)** Log-transformed coefficients of variation (CV) of MEP_single_ (red bars), MEP_double−1_ (light blue bars), and MEP_double−2_ (navy blue bars) at TMS_baseline−out_ and TMS_baseline−in_, elicited in the left or right first dorsal interosseous (FDI) muscle. Note that CVs were significantly reduced at TMS_baseline−in_ compared to TMS_baseline−out._ Also, MEPs were generally less variable in the right than in the left FDI; *p* ≤ 0.05 for both effects. **(B)** MEP_double−1_ and MEP_double−2_ CVs significantly fitted in windows ranging between 1.88 and 2.14 l.u. [log(100) = 2] indicating comparable CVs for all left and right FDI MEP_TYPE_. The vertical bars represent the smallest significant boundaries around the mean for each condition. Each plot refers to the above color-coded condition on the x-axis.

First, we focused on the CV of FDI MEPs elicited at TMS_baseline−out_ and TMS_baseline−in_ (Figure 5A). Overall, they equalled 54.8 ± 18.91% and 47.1 ± 17.88% at these two TMS timings, respectively. The ANOVA_RM_ revealed a significant effect of TMS_TIMING_ on the log-transformed data [*F*_(1, 13)_ = 5.14, *p* = 0.041], with smaller CVs at TMS_baseline−in_ than at TMS_baseline−out_. Hence, MEPs were generally larger and less variable when elicited at rest but in the context of a task, than when elicited outside the blocks. This effect tended to be stronger for MEPs elicited in the right than in the left FDI, but the TMS_TIMING_ x MEP_SIDE_ interaction did not reach significance [*F*_(1, 13)_ = 4.092, *p* = 0.064]. Though, the factor MEP_SIDE_ was significant [*F* = 7.67; *p* = 0.02]: CVs were smaller for MEPs elicited in the right FDI compared to when they were evoked in the left FDI, indicating an overall smaller variability of MEPs in the dominant hand. Importantly, all these effects occurred regardless of whether the MEPs were elicited using a single-coil or a double-coil_1 ms_ procedure. That is, neither the factor MEP_TYPE_ [*F*_(2, 26)_ = 0.049, *p* = 0.952], nor its interaction with the other factors (all *F* ≤ 1.431, all *p* ≥ 0.257) were significant. Similar to the MEP amplitudes, in order to assess the statistical bioequivalence of the double-coil_1 ms_ and single-coil CVs, we expressed the CVs of MEP_double−1_ and MEP_double−2_ as log-transformed percentages of MEP_single_ and tested whether these normalized data were significantly different from boundaries set at ±0.4 around 2. As we can see on Figure 5B, the MEP_double−1_ and MEP_double−2_ even fitted in smaller windows (all MEP_double−1_ between 1.88 and 2.14 l.u. [i.e., between 94 and 107% of MEP_single_] and all MEP_double−2_ between 1.90 and 2.14 l.u. [95–107%]; all *p* ≤ 0.05).

#### CV of FDI MEPs recorded at TMS_delay_

**Figure 6:**
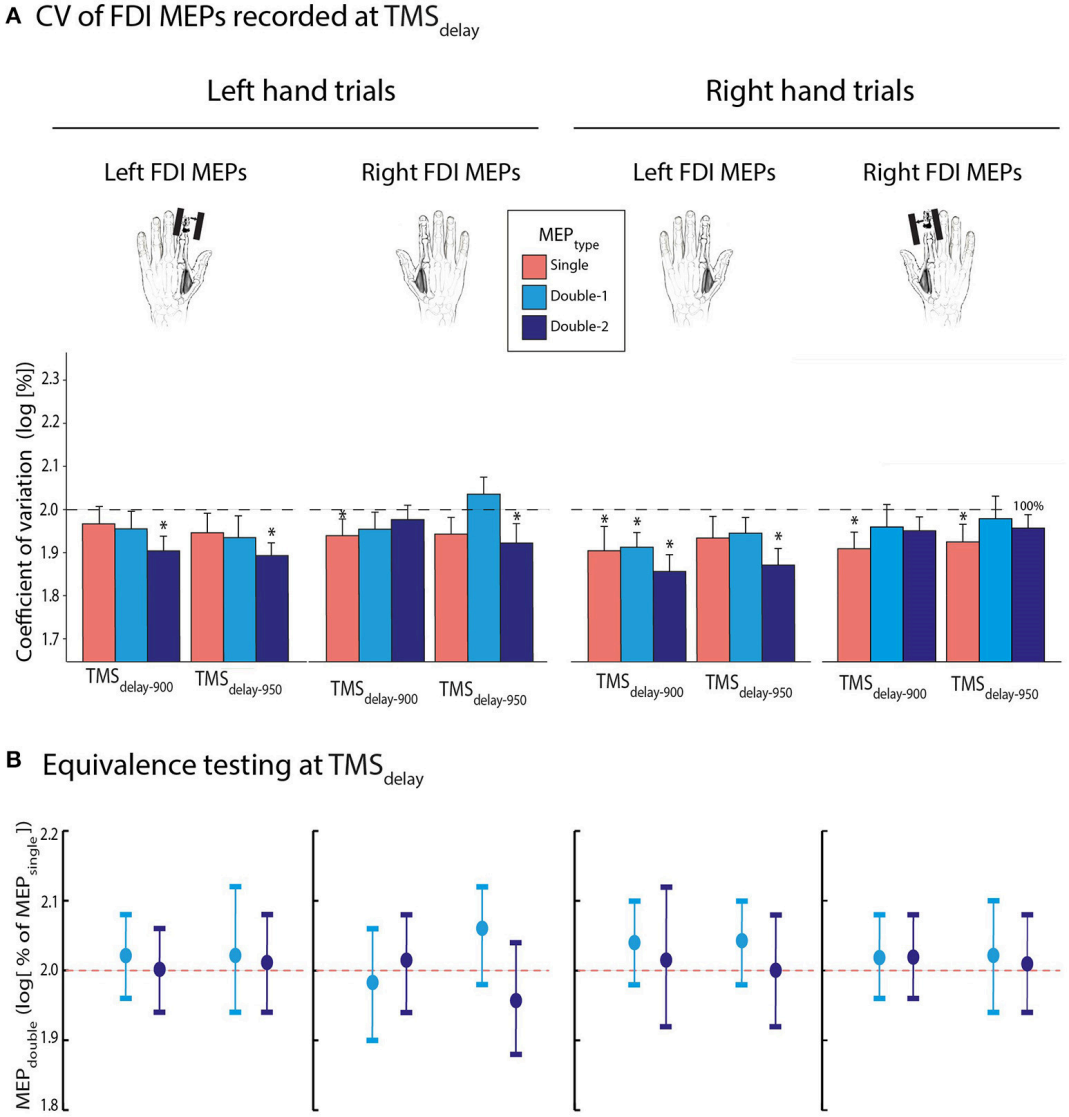
**(A)** Log-transformed coefficient of variation (CV) of MEP_single_ (red bars), MEP_double−1_ (light blue bars), and MEP_double−2_ (navy blue bars) at TMS_delay−900_ and TMS_delay−950_ (initially expressed as a percentage of TMS_baseline−in_), for the left or right first dorsal interosseous (FDI) muscles. Data are shown separately for MEPs acquired during left (left panel) and right hand (right panel) trials. Note that the factor MEP_TYPE_ was never significant. **(B)** Log-transformed CV of MEP_double−1_ and MEP_double−2_ at TMS_delay_ (initially expressed in percentage of MEP_single_). These data significantly fitted in windows ranging from 1.88 to 2.12 l.u. [i.e., between 94 and 106% of MEP_single_ in log], indicating comparable CVs for MEP_double_ and MEP_single_ during action preparation. The vertical bars represent the smallest significant boundaries around the mean for each condition. Each plot refers to the above color-coded condition on the x-axis. ^*^p ≤ 0.05.

Then, we turned to the CV of MEPs elicited during the delay period (Figure 6A). On average, they reached 90.2 ± 28.63 and 92.8 ± 36.12% of baseline values at TMS_delay−900_ and TMS_delay−950_, respectively. The *t*-tests performed on the log-transformed data revealed that CVs tended to show a further decrease at both TMS_delay_ timings compared to TMS_baseline−in_, although this effect was only significant for 37.5% of conditions; it was close to significance in 46.7% of the remaining conditions (0.05 ≤ *p* ≤ 0.10). The four-way ANOVA_RM_ did not reveal any further difference. None of the interactions or factors, including the MEP_TYPE_ [*F*_(2, 26)_ = 0.692, *p* = 0.509], were significant.

Again, at both delay timings, the log-transformed MEP_double−1_ and MEP_double−2_ data (initially expressed in percentage of MEP_single_) significantly fitted into a ±0.4 window around 2. As evident on Figure 6B, the MEP_double−1_ and MEP_double−2_ CVs even fitted in smaller windows (all MEP_double−1_ between 1.90 and 2.12 l.u. [i.e., between 95 and 106% of MEP_single_] and all MEP_double−2_ between 1.88 and 2.12 l.u. [94–106 %]; all *p* ≤ 0.05).

Hence, altogether, these data show that the double-coil_1 ms_ protocol is associated with comparable MEP amplitudes and CVs as the single-coil TMS procedure, whether these MEP parameters are assessed at rest or during action preparation.

#### Additional analyses on CV of ADM MEPs

The CVs were also computed for the ADM MEPs. Globally, we observed the same changes as those observed for the FDI. At rest, the CVs of ADM MEPs were globally smaller at TMS_baseline−in_ than TMS_baseline−out_ [*F*_(1, 13)_ = 18.314, *p* = 0.001] but comparable for the different MEP_TYPE_ [*F*_(2, 26)_ = 1.011, *p* = 0.378]. Consistently, the bioequivalence tests showed that all MEP_double−1_ and MEP_double−2_ amplitudes significantly fitted into smaller windows than ±0.4 (all MEP_double−1_ between 1.94 and 2.14 l.u. [97–107%] and all MEP_double−2_ between 1.92 and 2.20 l.u. [96–110%]; all *p* ≤ 0.05).

There was also a small trend for ADM CVs to decrease during the delay period with respect to TMS_baseline−in_ (but reaching significance in only 20.8% of TMS_delay_ conditions). Similarly to FDI CVs, the ANOVA_RM_ showed that CVs across the MEP_single_, MEP_double−1_, and MEP_double−2_ conditions were similar [*F*_(2, 26)_ = 1.284, *p* = 0.294]. No other interaction was found (all *F* ≤ 3.036, all *p* ≥ 0.105]. At both delay timings, all MEP_double−1_ and MEP_double−2_ CVs fitted in 1.94–2.12 [97–106 %] and 1.90–2.10 [95–105%] windows, respectively. Thus, MEPs elicited in an irrelevant muscle also displayed a CV that was comparable for the single-coil and double-coil_1 ms_ protocols.

## Discussion

### Summary of study goals

The goal of the present study was to assess whether the MEPs acquired using double-coil_1 ms_ are equivalent to those obtained by means of a classical single-coil TMS method. To do so, we compared MEPs elicited by a first (MEP_double−1_) or second (MEP_double−2_) double-coil_1 ms_ TMS pulse to MEPs obtained using single-coil TMS (MEP_single_). Both the amplitude and coefficient of variation (CV) of MEPs were considered. We compared these MEP variables in the context of a motor task requiring subjects to prepare and delay left or right index finger responses until the onset of an imperative signal. MEP_single_ are typically suppressed during the delay period (Bestmann and Duque, 2016; Duque et al., 2017). Here, we show that comparable inhibitory changes can be observed with MEP_double−1_ and MEP_double−2_. The MEPs exhibited comparable amplitudes and CVs, regardless of whether they had been elicited using a single- or double-coil_1 ms_ TMS approach.

### Comparing the amplitude of MEP_single_ and MEP_double_ during action preparation

The amplitude of MEPs was much smaller at TMS_delay_ compared to TMS_baseline−in_, consistent with many previous reports (Duque and Ivry, 2009; Greenhouse et al., 2015b; Lebon et al., 2016; Quoilin et al., 2016; Wilhelm et al., 2016). This effect was observed regardless of whether the MEPs were recorded from a muscle that was selected or non-selected for the forthcoming response. This result may seem inconsistent with previous work (Greenhouse et al., 2015a,b; Klein et al., 2016). However, several recent studies have failed to observe a difference of inhibition between selected and non-selected conditions, suggesting that this effect is not consistent and does not systematically show up (Quoilin et al., 2016; Wilhelm et al., 2016, 2017). As suggested in Quoilin et al. (2016), it is likely to depend on the task details, including the use (or not) of response devices, the presence (or not) of catch trials, the time at which TMS is delivered and eventually, the presentation of a feedback (or not). Inhibition at TMS_delay_ was also observed for a muscle that was irrelevant in the task, corroborating the idea that withholding a prepared action is associated with widespread inhibitory influences suppressing CSE until the movement can be initiated (reviewed in Duque et al., 2017). The suppression of MEPs tended to be deeper in the left compared to the right hand, consistent with the view that inhibitory changes are often more pronounced on the non-dominant compared to the dominant side (Leocani et al., 2000; Duque et al., 2007; Quoilin et al., 2016; Wilhelm et al., 2016). Note that this tendency was not observed in a previous work (Klein et al., 2016). Yet, an important difference there is that Klein et al. (2016) registered MEP_left_ and MEP_right_ in separate blocks, reducing the signal to noise ratio when comparing these conditions. Furthermore, we found that MEPs were similarly decreased at TMS_delay−900_ and TMS_delay−950_, probably because preparatory inhibition had reached a plateau by the time TMS was applied, in accordance with recent observations (Lebon et al., 2016).

Most importantly, the strength of the inhibitory effect at TMS_delay_ was comparable across all MEP_TYPE_. As such, bioequivalent analyses revealed that MEP_single_, MEP_double−1_, and MEP_double−2_ displayed the exact same level of suppression during action preparation. This result may stand at odds with another study in which we observed differences between MEP_single_ and MEP_double_ at TMS_delay_ (Wilhelm et al., 2016). However, an important weakness in that work is that the single and double-coil_1 ms_ protocols were tested in separate blocks. Hence, the difference between MEP_single_ and MEP_double_ was likely due to the fact that subjects were more vigilant or alert when they expected two pulses to occur (increasing MEP amplitudes) compared to when only one pulse was anticipated (Labruna et al., 2011a; Klein et al., 2012, 2014). By intermingling all conditions within each block, the present study allowed to control for this bias: our data show that in its absence, all MEP_TYPE_ display a comparable degree of suppression during action preparation. Note however that, because MEPs are rather global readouts of CSE, these results do not allow to rule out completely the occurrence of some bilateral interactions following double-coil_1 ms_ TMS. Yet, even if present, these interactions do not alter MEP amplitudes in a systematic way and do not preclude from obtaining measures of preparatory inhibition that are comparable to those acquired with single-coil TMS.

### Comparing the CV of MEP_single_ and MEP_double_ during action preparation

In order to evaluate changes in the variability of CSE during action preparation, we measured the CV of MEPs elicited using single-coil or double-coil_1 ms_ TMS. Overall, we observed a decrease in the CV of MEPs at TMS_delay_ compared to TMS_baseline−in_, even if this effect was not present in all conditions. Therefore, CSE tended to be less variable during action preparation compared to rest, consistent with a previous report (Klein-Flügge et al., 2013). Such a decrease in the variability of CSE during action preparation may reflect an optimization process of neuronal firing rates in the motor cortex (Churchland, 2006). Following this view, firing rates progressively become more consistent during action preparation, reaching a specific state to produce the desired movement (Rickert et al., 2009). Interestingly, this small decrease in the CV of MEPs at TMS_delay_ was not only observed for the FDI but also for the ADM. Hence, the variability of CSE decreased for both task-relevant and irrelevant muscles; the tuning of motor activity during action preparation may thus not be completely specific to the agonist effectors (Churchland et al., 2010; Klein-Flügge et al., 2013). Most importantly, changes in the CV from TMS_baseline−in_ to TMS_delay_ were equivalent for MEP_single_, MEP_double−1_ and MEP_double−2_, suggesting that double-coil_1 ms_ TMS is as effective as single-coil TMS to capture changes in the variability of CSE during action preparation.

### Comparing the amplitude and CV of MEP_single_ and MEP_double_ at rest

In the present study, we acquired two baseline measures of MEPs at rest. That is, MEPs were elicited during the intertrial interval (TMS_baseline−in_) within the blocks, but also outside the blocks (TMS_baseline−out_). At both timings, MEP amplitudes were generally comparable when elicited in the left or right hand, confirming that measures of CSE are highly comparable for both hemispheres at rest (Davidson and Tremblay, 2013). Yet, the CV of FDI MEPs was smaller in the right than in the left hand. Hence, neuronal firing rate may be steadier on the dominant side. Interestingly, MEP amplitudes were larger when acquired within the blocks compared to outside them and this effect was associated with a decrease in the CV of MEPs. Hence, CSE was larger and less variable when probed within the context of the motor task compared to when the subjects were at complete rest, outside the blocks. Such an effect on MEP amplitudes has been reported in a previous study comparing different baseline conditions (Labruna et al., 2011a). That is, Labruna et al. (2011a) showed that MEPs were larger when elicited in the context of a task requiring subjects to passively view hand or landscape pictures than when elicited outside the task, suggesting that the level of vigilance has a significant influence on CSE.

Importantly, our bioequivalence analyses revealed that MEP_single_, MEP_double−1_, and MEP_double−2_ were comparable in all baseline conditions. The bioequivalence of MEPs at complete rest (TMS_baseline−out_) had already been reported in a previous study (Grandjean et al., 2018). Here, we show that this equivalence persists when baseline MEPs are elicited in the context of a motor task (TMS_baseline−in_).

### Comparing the impact of MEP_single_ and MEP_double_ on reaction times (RTs)

First of all, RTs were generally faster with TMS_delay_ than with TMS_baseline−in_, consistent with many previous reports showing that a TMS pulse applied close to the imperative signal can prime subjects to respond faster (Duque et al., 2012; Labruna et al., 2014; Greenhouse et al., 2015b; Quoilin et al., 2016) probably because the TMS sound triggers the release of the movement that is being prepared (Carlsen et al., 2007, 2011). Interestingly, we also found that RTs were longer in trials where a MEP_single_ occurred in the responding hand compared to when the MEP_single_ fell in the non-responding hand, or in both hands at once (MEP_double_). This effect was present in all conditions at TMS_delay_, indicating that the boosting effect of the TMS sound was slightly attenuated when the MEP fell specifically in the responding hand, compared to when it fell in the other hand or in both hands, consistent with other works (Duque et al., 2013; Wilhelm et al., 2016). Surprisingly this effect of the MEP condition was also observed with TMS_baseline−in_ in right hand trials but not left hand trials. This result was unexpected given that here, MEPs were elicited during the intertrial interval and should thus not affect behavior, an issue for future investigation.

### Advantages of double-coil_1 ms_ TMS and future directions

The double-coil_1 ms_ protocol shows many advantages over the regular single-coil technique. First, the number of MEPs that can be collected in a given amount of time is doubled. This is a crucial aspect as it gives the opportunity to test more conditions within the same duration than could be done with a regular single-coil method. Second, CSE is probed bilaterally on the same trial meaning that both hands can be probed simultaneously. Hence, dominant and non-dominant hand MEPs are elicited in the exact same conditions during the task (Duque et al., 2013). This obviously increases the signal to noise ratio in a significant way. Third, the acquisition of MEPs in both hands allows researchers to make direct comparisons between bilateral MEPs on a single-trial basis and to develop new measures such as indexes reflecting the ratio between the CSE of the two hands. In fact, one may be interested in studying the impact of various task parameters (e.g., instruction, presence of reward, sensory evidence, level of urgency, effort required etc.) on the relationship between bilateral MEP amplitudes and CVs. Hence, the present technique opens new horizons in the study of how both hemispheres interact in various task settings (Verleger et al., 2009; Klein et al., 2016).

## Conclusion

The present study suggests that the double-coil_1 ms_ TMS can be used to probe CSE within the context of a motor task. As such, we show that MEPs elicited using a double-coil_1 ms_ technique are equivalent to those obtained by means of single-coil TMS, both at rest and during action preparation. This new method is promising since it allows to record MEPs from both hands simultaneously, doubling the amount of data that can be acquired in a given period of time. The development of double-coil_1 ms_ TMS might participate in the actual expansion of TMS in a broad range of neurophysiological as well as neurological studies.

## Author contributions

PV and JG: contributed equally; PV, JG, GD, and JD: designed research; PV, JG, YdW, and LQ: performed research; PV, JG, GD, YdW, LQ, and JD: analyzed data; PV and JD: wrote the article.

### Conflict of interest statement

The authors declare that the research was conducted in the absence of any commercial or financial relationships that could be construed as a potential conflict of interest.
